# Knowledge and Perceptions of Latent Tuberculosis Infection among Chinese Immigrants in a Canadian Urban Centre

**DOI:** 10.1155/2015/546042

**Published:** 2015-11-24

**Authors:** Jie Gao, Nicole S. Berry, Darlene Taylor, Scott A. Venners, Victoria J. Cook, Maureen Mayhew

**Affiliations:** ^1^Faculty of Health Sciences, Blusson Hall, Simon Fraser University, 8888 University Drive, Burnaby, BC, Canada V5A 1S6; ^2^Provincial TB Services, Clinical Prevention Services, BC Centre for Disease Control, 655 West 12th Avenue, Vancouver, BC, Canada V5Z 4R4; ^3^BC Women's Health Research Institute, BC Centre for Disease Control, Room H203F, 4500 Oak Street, Vancouver, BC, Canada V6H 3N1; ^4^School of Population and Public Health, University of British Columbia, 2206 E Mall, Vancouver, BC, Canada V6T 1Z9

## Abstract

*Background*. Since most tuberculosis (TB) cases in immigrants to British Columbia (BC), Canada, develop from latent TB infection (LTBI), treating immigrants for LTBI can contribute to the eradication of TB. However, adherence to LTBI treatment is a challenge that is influenced by knowledge and perceptions. This research explores Chinese immigrants' knowledge and perceptions towards LTBI in Greater Vancouver.* Methods*. This mixed methods study included a cross-sectional patient survey at BC's Provincial TB clinics and two focus group discussions (FGDs) with Chinese immigrants. Data from FGDs were coded and analyzed in Simplified Chinese. Codes, themes, and selected quotes were then translated into English.* Results*. The survey identified a mean basic knowledge score: 40.0% (95% CI: 38.3%, 41.7%). FGDs confirmed that Chinese immigrants' knowledge of LTBI was low, and they confused it with TB disease to the extent of experiencing LTBI associated stigma. Participants also expressed difficulties navigating the health system which impeded testing and treatment of LTBI. Online videos were the preferred format for receiving health information.* Conclusion*. We identified striking gaps in knowledge surrounding an LTBI diagnosis. Concerns of stigma may influence acceptance and adherence of LTBI treatment in Chinese immigrants. Integrating these findings into routine health care is recommended.

## 1. Introduction

In British Columbia (BC), Canada, the ethnic breakdown of people with tuberculosis (TB) has changed over the last several decades. Since 2010, there has been an average of 269 active TB cases per year in BC, more than 70 percent of which affected immigrants [1]. In 2013, the majority of TB cases were in Chinese-, Punjabi-, Tagalog-, Korean-, and Vietnamese-speaking immigrants, with the highest proportion of TB cases being immigrants from Mainland China [2]. Immigrants also accounted for the majority of persons starting treatment for latent TB infection (LTBI)—64.1 percent in 2011 [1]. Most active TB cases in immigrants developed from LTBI that had been acquired in their countries of origin based on genotyping studies [3–5]. The WHO has recommended treating LTBI in immigrants as a strategy that can further reduce active TB cases in low incidence settings such as BC [6].

Initiation of and adherence to LTBI treatment are generally low in immigrants to North America [7–9]. In Canada, drug therapy for LTBI is usually four to nine months depending upon the regimen used [5]. Incomplete courses of treatment are less effective in preventing TB disease; thus, adherence to and completion of the entire course of LTBI therapy are essential [10, 11]. Low treatment adherence among immigrants partially depends on their knowledge and perceptions of LTBI [12–16], which can be influenced by the use of culturally specific educational materials and cultural brokers [17, 18]. Providing accessible, culturally sensitive messaging through a variety of formats is hypothesized to increase rates of treatment initiation and consequently increase acceptance and completion of LTBI treatment [19, 20].

Because Chinese immigrants account for the highest proportion of TB cases in BC, the qualitative part of the study focused on Chinese immigrants [2]. Previous studies have identified some issues related to knowledge and perceptions of LTBI and its treatment among Chinese immigrants in North America [14, 16]. These studies focused on active TB and reported that Chinese immigrants' knowledge of the transmission of TB bacteria and prevention of TB disease was low. Chinese immigrants generally believed in Western health care providers' abilities and knowledge and were willing to seek care from them but preferred traditional Chinese medicine. Moreover, access to health insurance and the stigma associated with TB led to people not seeking medical care even if they suspected that they had TB [14, 16]. We found no studies that specifically examined perceptions of LTBI. The objectives of this research were to (a) document knowledge levels of patients in a TB clinic; (b) identify Chinese immigrants' knowledge and perceptions of LTBI; (c) specify messages that would be most appropriate for LTBI education in this population; and (d) identify the most appropriate format of health promotional materials to address the specific needs of this population.

## 2. Methods

As part of a larger TB education project that included developing culturally relevant and informative materials for a diverse group of immigrants, we conducted a mixed methods study. It included a patient survey at the Provincial TB clinics in Greater Vancouver that evaluated knowledge of LTBI and treatment of it [21] and a qualitative approach using FGD for Chinese immigrants [22, 23].

A survey in English of patients who visited the BC Centre for Disease Control (BCCDC) TB clinics was conducted from June 2013 to June 2014 [24]. Survey questions of knowledge, attitudes, and perceptions regarding LTBI and its treatment were developed and pilot-tested in five clerical staff from BCCDC to establish clarity of questions. To estimate the level of knowledge only very basic facts about LTBI were included in the survey, and we considered a score of 80% or higher as representing adequate knowledge. After refining them, the questions were verified again by the same five clerical staff. Since no further edits were required, we then pilot-tested the questions for clarity with 20 patients. No revisions were required, and these 20 surveys were excluded from the analysis. Respondents to the survey included all individuals who presented to the TB clinics and were 18 years old or older. Each respondent provided a unique identifier, which was used to exclude any duplicate surveys.

Using focus group discussions (FGDs), we then explored further details of knowledge and perceptions towards LTBI specifically in Chinese immigrants. Participants were purposively recruited from previously consented survey respondents [25]. Posted advertisements were used to recruit additional participants. All participants self-selected either early (well-settled) immigrants or recent (new) immigrants according to their self-perceived levels of adjusting to the new culture, their social engagement with Canadians, their perceptions of settlement, their feelings of belonging, and length of time residing in Canada [14, 26, 27].

Ethics approval was obtained from the Behavioural Research Ethics Board of the University of British Columbia and the Office of Research Ethics of Simon Fraser University. For survey respondents, consent was implied when they completed the survey. Survey respondents who provided their emails and/or phone numbers were contacted for FGDs. For FGDs, introductory letters and consent forms written in Simplified Chinese were sent to each participant through email two weeks prior to the FGDs. Signed consent forms were collected at the focus groups.

### 2.1. Data Collection

Surveys were distributed by trained clerical staff at the BCCDC TB clinics to all patients of 18 years old or older. Patients placed the completed surveys in a box at the TB clinic reception. Data were input weekly into a Microsoft Office Access database by a trained clinic clerk, reviewed for accuracy by a study team member, and then imported into a Microsoft Office Excel dataset for data analysis.

For FGDs, a structured topic guide, informed by the literature, was developed in English and then translated into Simplified Chinese. Four key questions guided the data collection: (1) What do you know about treatment options for latent TB infection? (2) What have you heard in your family or community about latent TB infection and about the treatment of it? (3) What might influence whether someone with latent TB infection takes treatment? (4) How would you interest your community in learning about how to prevent active TB from latent TB infection? This topic guide was pilot-tested in individual interviews and a mock FGD. The interviews were conducted in Mandarin with four Chinese volunteers recruited by one research assistant to ensure clarity of the topic guide questions. The topic guide was edited iteratively after each interview. A mock FGD conducted in English under the supervision of an experienced coinvestigator who did not speak Mandarin served to train two Mandarin-speaking research assistants as facilitators. The purpose of this training was to ensure consistent processes used by the focus group facilitators during FGDs.

Two FGDs were then conducted in Mandarin by the two facilitators—one as the moderator and a second as note taker. Prior to conducting the focus groups, a pamphlet was prepared describing basic facts about LTBI. It was distributed to participants after the first key question so that subsequent questions could be answered even if initial knowledge levels regarding LTBI had been low. The FGDs lasted between 1 and 2 hours. They were audiotaped and transcribed in Simplified Chinese by the moderator. Anonymity was preserved by assigning and using a unique number to identify each study participant.

### 2.2. Data Analysis

#### 2.2.1. Survey

All statistical analyses were executed with RStudio Version 3.0.3 for Windows (RStudio Inc., Boston, Massachusetts). Demographic information of survey respondents was calculated as proportions of total respondents, and a mean knowledge score was calculated from six equally weighted questions on basic facts about LTBI. Statistical significance was set to 0.05.

#### 2.2.2. FGDs

**Figure 1:**
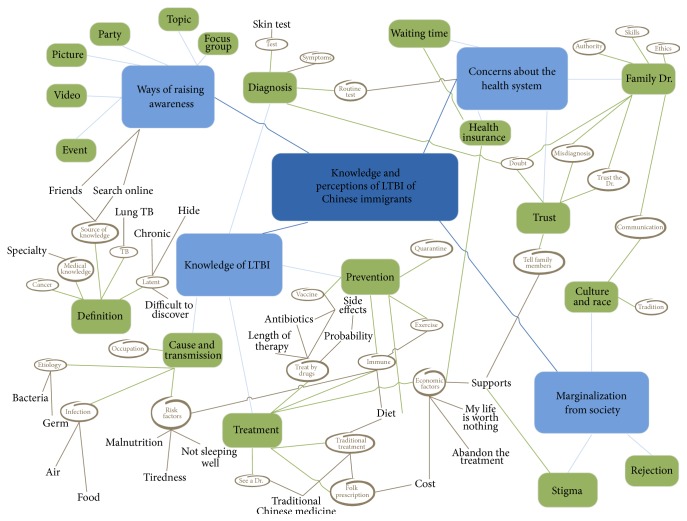
Codes from transcripts of the two focus group discussions.

Open coding of data from transcripts and field notes was performed in Simplified Chinese to preserve nuances during translation [28]. Codes, themes, and selected quotes that were particularly representative of the major themes were then translated into English. All transcripts were reviewed and coded by three Mandarin-speaking research assistants who sought to identify patterns and trends in emergent themes relevant to LTBI and its treatment. They began by independently reviewing the FGDs transcripts through line-by-line examination from which each research assistant identified relevant codes. The three coders then met to merge their codes into one code book. Coders identified overlap and disagreements among the codes. Codes were aggregated to decrease overlap, and disagreements were addressed by having the coders review the primary data together until consensus among them was reached. They continued to code the transcripts independently, consult with each other, and update the code book iteratively as necessary [29, 30]. All codes were used to create a conceptual map from which four major themes emerged (Figure 1). To establish themes, codes were grouped into subcategories and categories according to their meanings that were described in the code book, and lines were drawn to display the connections between codes and categories. The conceptual map was shared with our research team to ensure interpretations of the codes were meaningful, to discuss the themes, to relabel or regroup the data when necessary, and to determine if additional information needed to be solicited from the transcripts. Quotes were then sorted into each category and subcategory, followed by comparing and contrasting quotes within and between groups to identify differences and similarities. One research assistant documented the process and the themes that emerged [22, 23].

## 3. Results

### 3.1. Survey

Of 912 survey respondents, 67.1% were women, 91.7% had completed high school, and the largest age group among respondents was 25–44 years old (49.3%). Immigrants accounted for 71.2% of the respondents.

Only 19.8% answered that LTBI had no symptoms; 19.0% believed that LTBI did not spread from person to person; and 44.0% knew that treating LTBI prevented TB. Most patients (63.9%) answered that LTBI was treated with prescribed TB medicine, but fewer (41.4%) knew the duration of treatment. Nearly half of respondents (43.8%) answered that BCG vaccination conferred life-long immunity. The mean knowledge score was 40.0% (95% CI: 38.3%, 41.7%) for all respondents (Table 1).

### 3.2. Focus Group Discussions

All participants originated from Mainland China, and Mandarin was their native language. The first group included six early migrants who had immigrated to Canada more than six years ago: two females and four males with a mean age of 40.3 years old. Two-thirds had graduated from university. The second group included six recent migrants who had lived in Canada for six or fewer years and all were female with a mean age of 34.5 years old. Half had obtained a high school diploma (Table 2). Among the twenty-five potential participants contacted (sixteen survey respondents and nine people recruited through advertising), twelve participated in this study to completion. The remaining thirteen (all survey respondents) either did not respond (*n* = 8) or refused to participate (*n* = 5).

Four major themes emerged from the FGDs: knowledge of LTBI; concerns about the health system; marginalization from society; and ways of raising awareness (Figure 1).

#### 3.2.1. Knowledge of LTBI

Participants in both focus groups expressed low levels of general knowledge concerning LTBI (Figure 1).

*Definition*. All of the participants confused LTBI with active lung TB, and some recent migrants used the word “consumption” to represent LTBI and lung TB. Furthermore, two recent migrants considered LTBI to be a lethal cancer. 

*Cause and Transmission*. In each focus group, participants frequently talked about LTBI as being caused by either bacteria or, even more commonly, by a “germ.” Suggested risk factors for LTBI included “when the immune system is weak,” “malnutrition,” “tiredness,” and “not sleeping well.” One recent migrant believed that LTBI was an “occupational disease” caused by “breathing in small pieces of wood with chemical substances painted on it.”

Participants in each group knew that TB could be transmitted through the air from the cough of a person with active TB, and most of them explicitly stated that TB bacteria could affect anyone, particularly in public places. One recent immigrant indicated that LTBI could be transmitted through food because “food contains TB bacteria.” 

*Diagnosis*. Participants in each group described the signs and symptoms of LTBI as equivalent to the symptoms of active TB: “coughing,” “spitting up blood,” “weak,” and “becoming thinner and thinner” were all mentioned. No participant knew how to identify LTBI or where testing was available. Only one early immigrant said that there was a skin test used to diagnose “active TB” since she had previously undergone the test. She expressed concerns about the validity of the skin test:

*I told him [my family doctor] that I was given the vaccine before. So, there are antibodies in my body. But, the skin test may neutralize the antigens. So, the results will be false negative… So, I asked him if it was really necessary for me to do this skin test.*

*Prevention*. All early immigrants and most recent immigrants believed that one could prevent LTBI from developing into active TB by doing exercises and having a balanced diet, for example, “eating a lot of Vitamin C” in order to “strengthen the immune system.” In contrast, one recent migrant was less hopeful for patients with LTBI: “If you have LTBI, I think you can do nothing. You should really try your best not to develop other diseases. I mean, do not entice, do not trigger, or do not wake up the bacteria.”

*Treatment.* No participant knew which medications could be used to treat LTBI. After this information was provided to them, participants in both focus groups expressed concerns about taking medications: “the standard length of therapy for LTBI is too long,” “all the antibiotics have side effects, such as fungal infection,” “the cost of the medicines should not be too high,” and “the probability of developing active TB from LTBI is relatively low compared to side effects of the drugs.” In addition, “not having symptoms” and “not feeling sick” were also mentioned in both groups as reasons to refuse any treatment.

When they were asked which treatment(s) they considered the most effective, no consensus was reached. Some participants said traditional treatment methods were preferable while others disagreed and preferred to seek Western interventions. A few participants said they would consider traditional treatment only if “the doctor cannot cure my disease.” Traditional treatment included “traditional Chinese medicines,” “eating garlic,” “drinking one's urine,” and “heating and smelling white vinegar.”

The cost of the treatment was an important consideration for the majority of the participants and was mentioned in both groups. They worried that the costs of LTBI treatment were too high and represented a significant barrier to seeking care.

*For example, if I'm poor, and the folk prescription is relatively cheaper, I'll try it. My life is worth nothing anyway. But if I have the money, I'll go to see a medical expert, definitely… People who are rich won't abandon the treatment. *

#### 3.2.2. Concerns about the Health System

Participants in both groups discussed at length their concerns about the health system, which could hinder or encourage whether they sought health care for LTBI (Figure 1). 

*Family Doctor*. Participants in both groups stated that in general they trusted medical professionals in Canada to provide appropriate care. However, almost all participants questioned if their family doctors had enough knowledge of LTBI to diagnose this disease correctly. Moreover, cultural and language barriers were highlighted by early immigrants as barriers that hindered effective communication with family doctors.

*The family doctor should communicate with us using the language or expressions that we can understand. For example, they can tell us eating garlic may be good for your health, which could be easy for us to understand… We know that we Chinese all eat garlic. So, I mean, they need to use the language that is similar to our daily language so we can accept it more easily.*

*Waiting Time*. Most early migrants commented on the long waiting times associated with the Canadian health system and that decision-makers should make a concerted effort to address this. This comment was based on their experiences or those of their friends. They indicated that they might “go to other countries to treat the disease instead of waiting for a long time in Canada.”

#### 3.2.3. Marginalization from Society

All of the recent migrants and most of the early migrants believed that people with LTBI would be marginalized from society (Figure 1). 

*Rejection by Others*. Many participants voiced that people with LTBI might be rejected by their neighbours, relatives, or friends: “their first inclination will be to go away from me if I am diagnosed with LTBI.” 

*Stigma*. Stigma associated with LTBI was mentioned as a portrayal of self-marginalization. Some participants believed that their neighbours would panic and disclose a diagnosis of LTBI to others. They also worried that people might label them as sick persons who could transmit the disease.

*I'm afraid my neighbours will discriminate against me. They will tell everyone they know that I have this disease… Some of your friends will isolate you. They may think, ‘Oh, he/she has LTBI'… They won't believe me. Even if I tell my friends I have LTBI, and it is not infectious, will they believe me?*

In order to protect themselves from stigma, most participants said that they would keep it a secret if they were diagnosed with LTBI. They would choose not to tell their neighbours or friends because “it was not infectious.” Three early migrants disagreed, saying that if they had LTBI, it was their responsibility to tell their neighbours. One recent migrant also said that she would tell her neighbours because “my neighbour will see me taking drugs every day. So, I do not want them to guess what kind of disease I have.” All participants would inform their families since they felt their family members could provide support to them, including helping “figure out how to treat LTBI.”

#### 3.2.4. Ways of Raising Awareness

All of the participants were interested in learning about LTBI and its treatment and had various suggestions to increase Chinese immigrants' awareness. Ideas ranged from creating pamphlets to posting videos in Mandarin and Simplified Chinese on websites that were popular for Chinese communities including “Vansky,” “BCbay,” and Facebook (Figure 1).

## 4. Discussion

Although past research has generalized inquiries regarding LTBI from research on active TB, our results support the need to study LTBI as a separate and specific phenomenon. Chinese immigrants may be more likely to have negative attitudes towards LTBI treatment compared to TB treatment for several reasons. Treating active TB patients can eliminate symptoms in a short time while treating people with LTBI cannot improve their immediate well-being because LTBI causes no symptoms [9, 31]. Secondly, unlike active TB, LTBI is not contagious and thus the moral urgency of protecting others through one's own treatment is mitigated [31]. More importantly, participants in our study raised specific concerns about LTBI treatment in that “the probability of developing active TB from LTBI was relatively low compared to side effects of the drugs.” This concern regarding side effects has not been highlighted in studies of active TB probably because the perceived costs and benefits of taking medications to treat active TB are quite different from those of LTBI [14, 16].

Specific actions that can address each of our main findings are listed in Table 3. We identified the need for health promotional materials that are specifically tailored to addressing knowledge and perceptions of LTBI among the Chinese immigrant community. In our study, TB knowledge was very low for all survey respondents and FGDs participants. LTBI was often confused with TB disease, and this confusion may influence the demand for LTBI diagnosis and treatment, in that treatment may be considered unnecessary if LTBI is perceived as an incurable disease [32, 33]. Health educational materials such as videos or pamphlets with accurate, culturally relevant information on cause, transmission, diagnosis, treatment, and consequences of LTBI are required to address this knowledge gap [18, 34]. Health care providers should uncover how specific immigrant groups perceive LTBI treatment and provide them with materials to stress the distinction between LTBI and other diseases (Table 3). Such information may address misconceptions and barriers to treatment completion [13, 20].

Our study further uncovered several issues specifically related to Chinese immigrants' perceptions of the health care system that could also be addressed through health promotional materials. Lack of familiarity with navigating the health system and negotiating its inefficiencies were perceived barriers to being tested for LTBI and/or taking LTBI treatment. For example, study participants did not know where to seek health care for LTBI. Although medication for treating LTBI is free of charge in BC, many of the participants believed that cost was a barrier. They may be unable to or unwilling to seek health care because of these real and perceived barriers. To overcome these barriers, clear messaging on how to navigate the health system should be included in health educational materials. Providing incentives and assistance for Chinese immigrants could encourage treatment adherence [35]. To facilitate effective physician-patient communication, identified as a priority by early migrants, family physicians who provide health care to Chinese immigrants should be made aware of cultural differences in interpreting the meaning of LTBI and have access to culturally relevant health promotional tools, guidelines, or education and training programs (Table 3) [13].

Stigma related to LTBI arose in focus groups as a particularly important societal issue, and the reduction of stigma could be central to health promotion efforts in this area. A study on active TB conducted previously in Alberta, Canada, reported that many people in a local Chinese community knew that they should help others reduce TB-related fears [14]. However, in our study, none of the participants claimed to be willing to help others eliminate “LTBI-related panic” nor would they be open to receiving such help from others. Since strong social support can enhance adherence to treatment, stigma is likely to reduce LTBI treatment adherence. Patients may avoid seeking health care if they feel they must hide their LTBI diagnosis from their support networks [9]. To address this problem, community-based educational materials such as an online video, perceived as one of the most appropriate mediums by participants, could be created to raise awareness of LTBI community-wide and consequently influence adherence to LTBI treatment (Table 3) [13, 34].

*Strengths and Limitations*. This study met the criteria for rigor of qualitative research, namely, trustworthiness including credibility, transferability, dependability, and confirmability [36]. Credibility was achieved by the techniques of data triangulation and member checking. Data triangulation drew from the mock FGD, field notes, survey results, and discussions with the research team members. Member checking consisted of providing FGDs participants with interpreted themes and codes through emails and validation of our interpretation of data through individual phone calls by the same research assistant who facilitated the FGDs. To achieve transferability, our focus groups were assembled from both early and recent immigrants, as well as a diverse age range and mix of genders. This demographic diversity strengthened our ability to potentially capture knowledge and perceptions towards LTBI among immigrants who came from Mainland China to the Greater Vancouver area. A dependability audit was conducted by the primary investigator and one coinvestigator through reviewing the activities of the researchers in the whole process. Finally, a confirmability audit trail that included field notes, transcripts, and investigators' memos was used to provide linkages between the raw data and the final conceptualizations.

Our study had some limitations. First, our survey at the time of the focus groups was conducted in English, so even though Chinese immigrants frequently attended our clinic, few responded to the survey. The survey was later translated into Simplified Chinese. Secondly, due to limited resources, we were only able to conduct two FGDs, so thematic “saturation” may not have been achieved, and participants' perceptions may not represent Chinese immigrants' in rural parts of the province or in the rest of Canada [23]. Thirdly, we may not have captured all perspectives because we necessarily omitted the perspectives of any potential participants who refused to participate due to their beliefs or lack of time. Fourthly, the moderator of FGDs was a recent Chinese immigrant, and it is possible that early immigrants may not have identified with her. Finally, during data analysis, coders may have been focused on particular areas relevant to LTBI and could potentially have ignored others.

## 5. Conclusion

This study identified knowledge and perceptions of Chinese immigrants towards LTBI and highlighted key areas of educational efforts to focus on. It points to the importance of raising awareness of LTBI and reducing LTBI related stigma in Chinese immigrant communities. Communication barriers with family doctors could be addressed during routine health services for this patient population to encourage and promote increased adherence to LTBI treatment with a goal of further reducing TB incidence in BC. LTBI and TB diseases are distinct although related issues and our findings support studying them independently. Further research should include FGDs in other immigrant groups to ascertain if their perceptions are similar and evaluation of health promotional materials specific to immigrants to ensure that these materials overcome the barriers identified in this study.

## Figures and Tables

**Table 1:** Knowledge of LTBI from TB clinic survey (*N* = 912).

	*N*	%
What is the main symptom that indicates latent TB infection?		
Correct: no symptoms	181	19.8
Incorrect^1^	717	78.6
Missing	14	1.5
Can latent TB infection be spread from person to person?		
Correct: No	173	19.0
Incorrect	713	78.2
Missing	26	2.9
Latent TB infection can be treated with?		
Correct: Prescribed TB medicine	583	63.9
Incorrect^2^	316	34.6
Missing	13	1.4
The benefit of treating latent TB infection is?		
Correct: to prevent active TB disease	401	44.0
Incorrect	500	54.8
Missing	11	1.2
The treatment of latent TB infection lasts?		
Correct: 3–6 months/>6 months	378	41.4
Incorrect	517	56.7
Missing	17	1.9
Do you think BCG vaccine (a vaccine for TB) completely protects you from TB for your whole life?		
Correct: no	495	54.3
Incorrect	399	43.8
Missing	18	2.0

	Mean (%)	95% CI

Mean knowledge score	40.0	(38.3, 41.7)

^1^Incorrect answers included cough ≥3 weeks, cough with blood, fever, night sweats, weight loss, diarrhea, and do not know.

^2^Incorrect answers included general antibiotics, there is no treatment, herbal remedies, bed rest, acupuncture, and do not know.

**Table 2:** Characteristics of participants in focus group discussions.

	Early migrants (*N* = 6)	Recent migrants (*N* = 6)	Overall (*N* = 12)
	*N*	*N*	*N*
Age group			
18-19	1	0	1
20–29	1	3	4
30–39	0	2	2
40–49	2	0	2
51–57	2	1	3
Mean	40.3	34.5	37.3
Sex			
Male	4	0	4
Female	2	6	8
Education			
High school	1	3	4
College/trade school	1	2	3
University	2	1	3
Other (master's degree)	2	0	2
Working in the health care field			
Previously	2	0	2
No	4	6	10

**Table 3:** Learning objectives used to change knowledge and perception of LTBI among Chinese immigrants.

Findings		Impact on adherence	Interventions
*Perceptions of LTBI*			
Definition	What is LTBI	Hinders	Provide correct information about LTBI.
Cause and transmission	The risks of contracting LTBI	Facilitates	Make patient-perceived susceptibility more consistent with an individual's actual risk.
Diagnosis	Knowledge about the absence of symptoms and tests for LTBI	Hinders	Provide information on symptoms of TB and contrast with the absence of LTBI symptoms. Describe where the tests are available.
Prevention	The benefits of prevention to reduce risk of LTBI	Facilitates	Educate patients about the benefits of preventing active TB from LTBI and what action can be taken.
Treatment	Concerns about taking medicines: length of treatment, side effects, and costs	Hinders	Correction of the misinformation on side effects and costs; provide incentives and assist patients with treatment completion.
	Taking any treatment in the absence of symptoms	Hinders	Educate patients that having no symptoms does not mean there is no LTBI.
	Belief in some traditional treatment	Hinders	Replacing traditional treatment with standard LTBI treatment and potentially encourage harmless traditional treatments when that approach is beneficial.
	Benefits of LTBI treatment	Facilitates	Specify the positive effects of treating LTBI and what actions to take.
	Confidence in one's ability to take action to support for LTBI treatment completion: costs, jobs, financial resources, health insurance, and being familiar with the environment	Hinders	Inform patients that there is no financial burden for the treatment of LTBI; provide support to patients, including reminding patients to take medicine, counseling to reduce their anxiety, and providing opportunity for clinic hours that coincide with patient availability.

*Concerns of the health system*			
Health insurance	Barriers to adhering to treatment because of the health system: costs	Hinders	Inform patients that the treatment of LTBI is free and help them navigate the health care system.
Family doctor	Barriers to adhering to treatment because of the family doctor: knowledge, authority, and communication	Hinders	Reassure patients that family doctors are qualified to diagnose and treat LTBI and increase their communication skills through education or training programs. Provide easy-to-use tools to family doctors to help portray information more efficiently.
Waiting time	Barriers to adhering to treatment because of the health system: efficiency	Hinders	Improve the efficiency of the health system through strengthening the partnership between the primary health care system and TB services.
Trust	Benefits of trusting family doctors: good skills and ethics	Facilitates	Educate patients about the positive effects of trusting family doctors and adhering to their prescriptions.
	Barriers to adhering to treatment because of the family doctor: perception that family doctors use drugs as a test	Hinders	Educate immigrants on the rules of Canadian medical practice and how using medication for inappropriate reasons is not acceptable nor is it tolerated. Explain that a trial of treatment can be acceptable practice.

*Marginalization from society*			
Rejection by others	Barriers to adhering to treatment: rejection by others	Hinders	Increase social awareness and acceptance of LTBI.
Stigma	Barriers to adhering to treatment: a diagnosis of LTBI would become known to others	Hinders	Reduce the stigma of LTBI patients at a community level through community-wide educational interventions.
Culture and race	Barriers to adhering to treatment: rejection by communities	Hinders	Be aware of the cultural perceptions and increase the awareness and acceptance of LTBI in Chinese communities.

*Ways of raising awareness*			
	Strategies to activate “readiness”	Facilitates	Create, test, and evaluate health promotional materials to raise awareness.

## Appendix

See Tables 1, 2, and 3 and Figure 1.
